# Verbascoside-Rich Plant Extracts in Animal Nutrition

**DOI:** 10.3390/antiox13010039

**Published:** 2023-12-24

**Authors:** Raffaella Rossi, Edda Mainardi, Francesco Vizzarri, Carlo Corino

**Affiliations:** 1 Department of Veterinary Medicine and Animal Science, Università Degli Studi di Milano, Via Dell’Università 6, 26900 Lodi, Italy; edda.mainardi@unimi.it (E.M.); carlo.corino@unimi.it (C.C.); 2 National Agricultural and Food Centre Nitra, Hlohovecká 2, 95141 Lužianky, Slovakia; francesco.vizzarri@nppc.sk

**Keywords:** acteoside, animal health, nutrition, verbascoside, antioxidant status, meat quality

## Abstract

In recent years, the search for dietary intervention with natural products able to sustain animal health and decrease environmental impact, has raised the number of studies pertaining to the use of plants’ secondary metabolites. In fact, in livestock, there is a clear relationship between the animals’ antioxidant status and the onset of some diseases that negatively affect animal welfare, health, and productive performance. An interesting compound that belongs to the secondary metabolites family of plants, named phenylpropanoids, is verbascoside. The genus *Verbascum*, which includes more than 233 plant species, is the genus in which this compound was first identified, but it has also been found in other plant extracts. Verbascoside exhibits several properties such as antioxidant, anti-inflammatory, chemopreventive, and neuroprotective properties, that have been evaluated mainly in in vitro studies for human health. The present work reviews the literature on the dietary integration of plant extracts containing verbascoside in livestock. The effects of dietary plant extracts containing verbascoside on the productive performance, antioxidant status, blood parameters, and meat quality in several animal species were evaluated. The present data point out that dietary plant extracts containing verbascoside appear to be a favorable dietary intervention to enhance health, antioxidant status, and product quality in livestock.

## 1. Introduction

The secondary metabolites of plants or their phytochemical metabolites are essential to enable plants to survive in the environment. These organic compounds are generally bioactive in the host plant and play a role in its growth and reproduction, may play a role in herbivore prevention, and may act as antimicrobials, thus having an important task in maintaining the plant’s defense system [1,2]. Secondary metabolites comprise phenolics, terpenoids, alkaloids, flavonoids, and glycosides that present several therapeutic activities [3].

Phenolic compounds can be defined as any compound having a benzene ring with one (phenol) or more (polyphenol) hydroxyl groups [4]. As a wide group of bioactive chemicals, they perform several biological functions [5]. Among the numerous bioactive molecules, polyphenols are recognized as an important food and feed source with an extremely heterogeneous composition. They are present in different parts of plants, including roots, leaves, flowers, fruits, and seeds [6].

**Figure 1:**
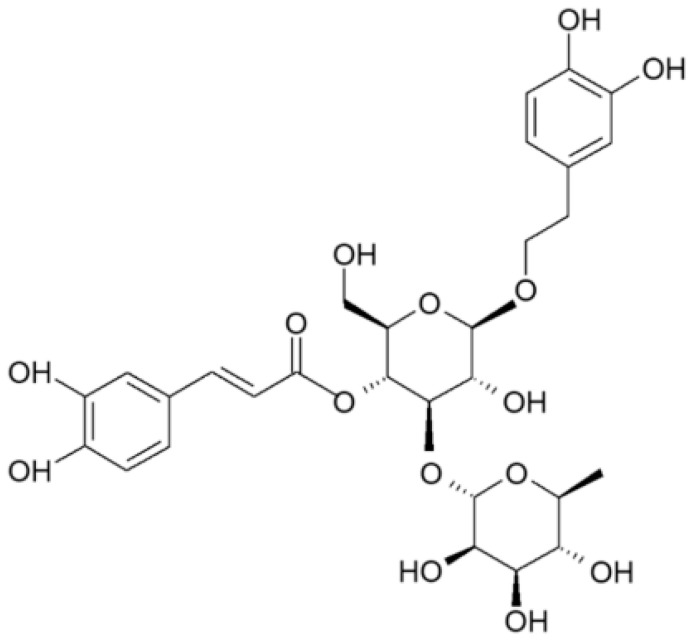
Chemical structure of Verbascoside. Phenyletanoid and caffeic acid bound to α-rhamnopyranosyl-β-glucopyranose.

Verbascoside (VB), also named acteoside, refers to a secondary metabolite family of plants called phenylethanoid glycosides. It was firstly identified in *Verbascum sinuatum* L. [7], but the purification and identification of VB dates to 1968 and it was identified as an acteoside, as reported in Figure 1 [8]. Verbascoside is the most prevalent of the disaccharide caffeoyl esters, found in different parts (primary and secondary root, leaves and flower) of several plant species. Until now, VB has been identified in the *Verbascum* species [9], and it has also been discovered in over 200 plant species such as *Oleaceae*,* Bignoniaceae*,* Verbenaceae*, and *Labiatae* [10,11]. Also, some industrial food wastes, such as olive mill wastewater, are considered important sources of VB [12,13].

Studies on the pharmacokinetics of dietary VB showed that it is subject to modification during absorption via methylation, sulfation, and glucuronidation in the small intestine and later in the liver [14]. A small amount of VB is absorbed into the cardiovascular system. In rats, after oral supplementation with 100 mg/kg of VB, a maximum serum concentration of 0.13 μg/mL and a half-life of 92.1 min was observed, with bioavailability of 0.12% [15]. In Beagle dogs, after oral supplementation with 10 mg/kg the absolute bioavailability is about 4%, with a maximum serum concentration of 0.42 μg/mL [16].

The low intestinal absorption of dietary polyphenols was observed due to their molecular sizes, degrees of polymerization and solubilities. Verbascoside, as a caffeic acid conjugate, passed poorly through the intestinal epithelial cells and its metabolites hydroxytyrosol and 3-hydroxyphenylpropionic acid are responsible for its activity. It is also observed that intestinal bacteria had an important influence on VB absorption and metabolism [17]. The main metabolic pathway of VB by intestinal bacteria seems to be hydrolyzation. One in vitro study reported that VB, incubated with intestinal bacteria or enzyme, generate eight degradation metabolites, two isomers, and four parent metabolites [18].

A previous study in rats showed that methylation is the primary form of metabolic degradation of VB and the final concentration of VB was low if compared with its metabolites. In this study, 19 metabolites of VB and 16 metabolites of its degradation were identified. Another study showed that 44 metabolites were identified in rats that received VB, and 35 were VB parent compounds [19]. So, VB works as a precursor of several active compounds and this explains why the oral bioavailability of VB was low [20].

Few studies have reported toxicological data on VB. The maximum tolerance dose of VB reported in humans was 0.443 mg/kg/day [21]. Moreover, previous studies have showed that the median lethal dose (LD50) of the aqueous extract of *Lippia Citrodora* leaves and verbascoside was higher than 5 g/kg in acute toxicity trials. No pathological, hematological, or biochemical toxic effect was reported with a sub-acute dosage in a 21-day trial in rats [22,23]. In an in vitro study on human fibroblasts, ethanolic extract of *Lippia citriodora* leaves (21% of VB) did not show any cytotoxic effects and to some extent exerted a cytoprotective effect against oxidative stress [24].

Several studies on plant extracts containing VB or on the pure compound have been conducted to assess its various biological activities and safety data. In vitro and in vivo studies reported that VB can be used in feed and food additives for improving the health and welfare of animals, product quality, and human health.

## 2. Biological Effects of Verbascoside

### 2.1. Antioxidant Activity

Oxidative stress, leading to the alteration of biological macromolecules such as lipids, proteins, and nucleic acids, is considered one of the causes in the pathogenesis of several diseases and aging [25]. Among the endogenous protective mechanisms that comprise the scavenging of reactive oxygen species (ROS), prevention of ROS formation, and enzymatic and non-enzymatic defenses, also the exogenous antioxidants such as polyphenols, provided from the diet, play a key role in the antioxidant system.

The antioxidant activities of VB, analyzed using different chemical laboratory methods in several experiments, are extensively reported in a review by Alipieva et al. [26]. Several assays were employed, such as Trolox-equivalent antioxidant capacity (TEAC), oxygen radical absorbance capacity (ORAC), hydroxyl radical averting capacity (HORAC), ferric reducing–antioxidant power (FRAP), and the CUPRAC assay. It reported the VB capacity for H_2_O_2_ scavenging ability, superoxide anion (O_2_^−^) radical scavenging activity, nitric oxide radical scavenging activity, peroxy-nitrite radical scavenging activity, and DPPH radical scavenging activity.

It has been reported that VB shows direct ROS scavenging activity higher than ascorbic acid, and this is due to a free hydroxyl group in the glucose moiety found within its structure [14,27]. In fact, it has been reported that VB shows greater DPPH scavenging activity (IC_50_ 58.1 µM ± 0.6) than those reported for ascorbic acid, used a reference substance (284.9 µM ± 1.2). The VB hydroxyl radical scavenging capacity was 357 ± 16.8 µM and IC_50_ 1031 ± 19.9 µM for ascorbic acid [28]. Further studies reported that the antioxidant mechanism of VB is related to the gene transcription of antioxidant enzymes such as catalase, glutathione peroxidase, and glutathione reductase through a Nrf2-dependent mechanism [29,30].

Similarly, in human retinal endothelial cells treated with a high dosage of glucose, VB in the cells culture media at 50 µM can decrease the amount of ROS by 25% [31]. Moreover, the antioxidant activity of natural extracts containing VB (485 mg/100 g dry weight) was also dependent on its ability in chelate metal ions [32]. It has also been reported that VB passes the blood–brain barrier, preventing ROS production, preserving the antioxidant system and reducing advanced glycation end (AGE) products and 8-hydroxy-2′-deoxyguanosine [33,34]. The antioxidant activity of VB has also been observed on oocytes during in vitro maturation, as reported in a recent review by Xiao et al. [15]. It is, however, important to point out that in vitro studies of VB at different dosages and types of administration do not provide reliable information about its in vivo activities. However, these data may be useful in order to test the VB in in vivo studies on animals in order to define the proper dosage in relation to animal species and physiological phases.

### 2.2. Anti-inflammatory Activity

The anti-inflammatory properties of VB are related to the reduction in the production of superoxide radicals and inducible nitric oxide synthase (iNOS) expression, which is a crucial mediator of acute or chronic inflammation. It has been reported that VB suppresses the activator protein-1 (AP-1) that is essential for iNOS induction.

The anti-inflammatory effect of VB has also been observed in rats, with induced osteoarthritis, with a reduction in the activation of the nuclear factor-κB (NF-κB), which are a family of inducible transcription factors that regulate genes implicated in inflammatory responses [35]. It has also been reported that NF-κB is stimulated by ROS, and in this way, a strong relationship between oxidative stress and inflammation is highlighted [36].

Moreover, in rats treated with VB, a lower production of tumor necrosis factor (TNF)-α and proinflammatory interleukin (IL-1β, IL-6) has also been observed [35]. Consequently, in A549 cells, a lower apoptosis rate and apoptosis-related genes expression (Caspase 3, Caspase 8, and Caspase 9) has been observed. Also, in dendritic cells treated with VB, a lower production of proinflammatory cytokines, including interleukin (IL)-12 and TNF-α, and a high production of the anti-inflammatory cytokine IL-10 has been observed [37].

It has also been reported that the anti-inflammatory properties of VB are also related to the inhibition of the activity of the Cytosolic Ca2+-dependent phospholipase A2 (cPLA2) that reduces the release of arachidonic acid [38].

Even if in vitro studies are not sufficient to establish the in vivo anti-inflammatory effects and dosages of VB, it is important to have some information about the mechanism of action of VB to better define the physiological phase in which there are acute or chronic inflammatory phenomena and in which the VB can exert its activity. In fact, with inflammatory phenomena there is inadequate nutrient intake, due to increased energy, aminoiacids, and trace element requirements for increased basal energy expenditure, increased muscle catabolism, and increased anabolism for the synthesis of the several constituents of the immune system [39]. So, considering the anti-inflammatory effects of VB, it should be considered as a sustainable dietary strategy for the modulation of inflammatory phenomena in different animal production phases.

### 2.3. Antibacterial Activity

In recent years, many approaches have been studied to counteract bacterial resistance. In particular, attention has been focused on natural compounds, especially polyphenols, novel molecules with antibacterial activities [40,41]. This approach has included determination of the minimum inhibitory concentration (MIC) against pathogenic bacteria, biofilm formation assays, and transcriptional analyses.

One previous study has reported that a plant extract rich in VB showed antibacterial activities against *Staphylococcus aureus* [42]. The efficacy of ethanolic extract of Lemon verbena against the Staphylococcus aureus skin infection was evaluated in an in vitro and in vivo trial in mice. The mechanism of action is not fully elucidated but could be related to the modulation of the cell’s membrane-dependent processes. A recent study reported that an extract from *Stachytarpheta indica* leaves containing VB and isoverbascoside, an isomer of VB, exhibited strong antibacterial activity, determined by MIC, against three Gram-positive bacteria, *Staphylococcus aureus*, methicillin-resistant *Staphylococcus aureus*, and *Staphylococcus epidermidis* [43]. It has also recently been reported that the two active compounds extracted from the genus *Globularia* possessed a high antimicrobial activity against *Staphylococcus*, but a lower inhibitory activity against *Enterococcus faecalis*,* Escherichia coli*, and *Pseudomonas aeruginosa* in in vitro assays [44].

Recently it has also been observed in an in vitro study that *Lemon verbena* extract, containing VB, in combination with gentamicin, was effective against drug-resistant *Staphylococcus aureus* and *Escherichia coli* if compared with the antibiotic alone [45].

The antimicrobial activity of VB is related to several processes such as alteration of cell membrane permeability, elimination of the biofilm, and changes in cell morphology. So, the use of VB should be considered for its antimicrobial activity to prevent the microbial contamination of meat intended for fresh consumption [46]. In this study, three dosages of VB were sprayed on the surface of fresh meat samples (2 mL of solution contained 0.625, 1.25 or 2.5 mg/mL VB) stored at 4 °C for 9 days. With the highest dosage of VB, inhibition of spoilage bacteria growth and reduction in the total microbial level in the meat were observed. The effective dosage of VB employed in this study was lower than the reference dose established for humans [21].

### 2.4. Other Biological Activities

VB also presents other relevant biological activities such as antidiabetic, anticancer, hepatoprotection, renal protection, and a neuroprotective role in Alzheimer’s disease (AD), as reviewed by Xiao et al. [15]. All of these activities, tested in in vitro studies and in studies on mice and rats, are mainly linked with VB’s antioxidant and anti-inflammatory properties.

VB’s antidiabetic activity is principally related to carbohydrate digestion, decreasing α-amylase activity and glucose adsorption, negatively modulating the activity of the sodium-dependent glucose cotransporter 1, as observed in in vitro studies [47,48].

VB’s anticancer activity has recently been reviewed by Kan et al. [49], who reported its anti-proliferation, anti-angiogenesis, and pro-apoptotic effects in several types of cancer cell lines.

Moreover, it has been also observed that VB influences the AD onset and progression via neuroinflammation prevention, also modifying AD memory decline and learning disorders [50,51]. In eight-month-old male mice, expressing mutation associated with early-onset Alzheimer’s disease, fed for 6 weeks with 10 mg/kg of VB, a suppression of pro-inflammatory cytokine production and upregulation of anti-inflammatory ones was observed. Moreover, VB can block microglia and astrocyte against activation by regulating the NF-κB-p65 pathway [50]. The same VB dosage was fed for 42 days to transgenic male mice and attenuated endoplasmic reticulum stress and prevented apoptosis [51].

Recently, it was observed that intragastrical VB (20 mg/kg of body weight) from *Leucophyllum frutescens* extract, administered for 4 days, can preserve the integrity of liver cell membranes and lower blood concentration of aspartate aminotransferase and alanine aminotransferase in rats with induced necrotic damage. However, the mechanism of action of VB on liver protection is still not completely understood [52].

## 3. Verbascoside-Rich Plant Extracts in Animal Nutrition

In recent years, there has been increased attention on employing natural feed additives in livestock, for the presence of several secondary plant metabolites that can positively affect animal health and production [53]. In fact, in livestock, antioxidant status could be negatively affected by several biological, nutritional, and environmental stressors leading to the development of many diseases and chronic pathological conditions that negatively affect health and productivity [54,55,56].

High demands for milk and meat production from both ruminants and monogastric animals, raises the metabolism and consequently induces high ROS production that results in the impairment of the antioxidant–pro-oxidant balance [57,58].

In recent years considerable interest has also been focused on the effects of heat stress on animal health, due to the high increase in the global environmental temperature. Heat stress causes a reduction in feed intake, with a lower intake of antioxidant molecules, a reduction in the concentration of some antioxidant vitamins (vitamin E and vitamin A), and high ROS production with the activation of the heat shock protein pathway [59]. Moreover, it has also been observed that acute or chronic heat stress can negatively affect quality parameters, oxidative stability, and the shelf life of meat and derived products [60,61].

For these reasons, the employment of natural extracts containing polyphenols has increased as a feed additive because they are perceived as sustainable if they are compared with synthetic substances. Considering the numerous biological activities of VB, previously described, its application in animal nutrition seems to be promising for improved health, productive parameters, antioxidant status, and product quality.

In the literature, dietary supplementation with *Lippia citriodora* extract, rich in VB, has been widely employed in livestock. The polyphenol content of *Lippia citriodora* extract is reported in Table 1 [62].

### 3.1. Monogastric Animals

#### 3.1.1. Pig

Some data have reported the effects on pigs of dietary supplementation with plant extracts containing VB (5 and 10 mg/kg of feed), affecting growth performance, antioxidant status, and meat and product quality parameters.

In swine production, the weaning period is very critical for piglet health. The separation from the sow and the change in the diet from milk-based to a complex plant-based diet, causes a lot of stress that reduces feed intake and negatively affects the growth and health of piglets. In addition, inflammation phenomena affect intestinal permeability with a negative effect on nutrient absorption and the onset of post weaning diarrhea (PWD), which negatively affect morbidity and mortality [63]. Moreover, oxidative stress usually occurs and is related to various environmental and physiological factors, which impair antioxidants status, immune parameters, and growth performance [56]. So, dietary interventions with several natural antioxidant substances have been reported as sustaining these parameters [64,65,66].

In post weaning piglets, dietary supplementation of feed for for 52 days with *Verbenaceae* leaf extracts, which contain 5 and 10 mg/kg of feed of verbascoside, was studied [67]. The result showed that a high dosage of dietary *Verbenaceae* leaf extract improved the piglets’ average daily gain (ADG) and tended to improve the piglets’ final body weight. In the same study, a high antioxidant status, measured as serum concentrations of reactive oxygen metabolites and serum immunoglobulin A concentration, was observed in both groups fed with the natural extract and the control. Another study in post-weaning piglets fed *Verbenaceae* leaf extract, containing 5 mg/kg of verbascoside for 39 days, no effects on growth performance were observed, but higher blood antioxidant activity and a lower haptoglobin level than the control groups were reported [68].

The effects of dietary VB (5 mg/kg of feed) in post weaning piglets submitted to oxidative stress status induced by a high intake of omega-6 polyunsaturated fatty acid was also studied [62,69]. This dosage of VB can positively affect liver responses to oxidative stress, reducing heat shock protein 70 (HSP 70) expression without an effect on systemic antioxidant status [62]. Dietary VB protected the intestinal villi from oxidative damage and the intestinal morphology was preserved. The duodenum and jejunum villi height and villi-to-crypts ratio was observed, and the duodenum mucin layer was similar to the control group. No effects of dietary VB on gut taste receptors were also observed [68].

Considering these activities, the *Verbenaceae* leaf extract containing VB could be used in the post-weaning phase to have a protective role on liver and small intestine morphology, sustaining antioxidant status and consequently the piglets’ health.

Another study reported that long term dietary supplementation with *Verbenaceae* leaf extract, containing 5 mg/kg of feed of VB, from weaning to slaughter (166 days) did not affect growth performance but total blood antioxidant activity, measured with the biological test KRL, tended to be higher in pigs fed VB [67]. Even if long term dietary supplementation with VB did not affect the total antioxidant activity of pig blood at slaughter, it affected the intestinal mucosa antioxidant status, decreasing duodenum nitrotyrosine levels [69]. Probably the dosage of VB can exert a local antioxidant activity in the duodenal mucosa, decreasing the oxidative markers in pig gut and indirectly modulating the efficiency of gut barriers. In fact, a recent study reported that dietary supplementation with verbascoside for 12 weeks (100–150 mg/kg/day) in mice modulated the gut microbiota diversity, reducing intestinal dysbiosis and lowering intestinal barrier disruption, lessening inflammatory markers [70]. The improvement of gut health due to VB dietary supplementation seems to be helpful for its ability to sustain gut antioxidant status, enhancing pig health and subsequently decreasing the use of antimicrobials.

The effects of dietary VB on carcass characteristics and pork quality parameters were also evaluated [71,72,73,74]. In fact, dietary supplementation with antioxidant molecules is one approach to improve shelf life parameters, decreasing oxidative phenomena. The effects of long-term dietary integration of natural extracts, containing 5 mg/kg of feed of VB, on pork quality was investigated. An improvement in the oxidative stability of *Longissimus dorsi* (LD) muscle was observed (−96.2% compared to the control), together with the increased muscle content of α-tocopherol (+34.3% compared to the control) [73]. Moreover, an improvement in the sensory profile of the LD muscle was observed with a reduction in fat and rancid odor in cooked and refrigerated pork muscle.

Then, antioxidant mixture, in a daily amount of 150 mg of vitamin E and 15 mg of VB, was administered to pigs for 38 days before slaughter till they reached a slaughter weight of 135 kg [72]. The antioxidant mixture improved LD muscle color indexes and oxidative stability during eight days of refrigerated storage. The same mixture, administered in pigs for 45 days before slaughter, improved the color and oxidative stability and decreased *Pseudomonas* spp. counts in LD muscle packaged under a modified atmosphere for 21 days. Moreover, at 15 d of storage, the sensory shelf life revealed that muscle from pigs fed an antioxidant mixture presented the same appearance and aroma as fresh meat [73].

The effects of a dietary supplement containing VB was also evaluated in products derived from pork [74,75]. In salami Cremona, the color parameters and the fatty acid composition was improved by VB supplementation (5 mg/kg of feed) in pigs for 30 days before slaughter at 160 kg of live weight [74]. In the Speck, smoked dry-cured ham, derived from pigs fed an antioxidant mixture (vitamin E and VB) for 45 days, a lower seasoning loss with slight effects on volatile compounds and sensory attributes was reported. However, the consumer test showed that dietary VB raised the overall preference for Speck, possibly increasing its consumption [75].

In the literature, the stabilizing effects of dietary polyphenols on oxidative markers, color indexes and the sensory shelf life of muscle were observed, with an enhancement of muscle α-tocopherol content [76]. Considering these effects, dietary VB from *Verbenaceae* leaf extract seems a promising antioxidant molecule that positively affects pork quality and nutritional, technological, and sensory meat quality parameters.

#### 3.1.2. Hares

Some studies have shown the effects of dietary plant extracts, containing VB, in weaned hares, affecting growth performance, antioxidant status, and meat quality. In hares (*Lepus corsicanus*), two different dosages (5 or 10 mg/kg of feed) of a water-soluble extract of *Verbenaceae* (*Lippia* spp.) were supplemented for 62 days to evaluate growth performance and blood parameters [77]. An improvement in the average daily gain of the animals was observed. An improvement in lipid blood values (triglycerides −9.5 than control; HDL cholesterol +21.4 than control) was also reported. It has been reported that polyphenols may activate the peroxisome proliferator-activated receptor (PPARα), altering the expression of key proteins involved in the lipid metabolism. Moreover, an enhancement of the weaned hares’ antioxidant status was observed with high plasma levels of vitamin E (+15.3% over the control) and lower production of reactive oxygen metabolites (ROMs; −37.2% compared to the control), and thiobarbituric acid reactive substances (TBARS; −35.1% compared to the control). The increase in serum levels of vitamins can be related to the antioxidant activity of VB-rich plant extract in supporting the endogenous antioxidant system and decreasing oxidative stress markers.

It is possible that, after weaning, dietary supplementation with natural extracts containing VB can improve hematological profile and antioxidant status in the growing leverets with a positive effect on health and growth parameters [77].

Three different dosages of *Lippia citriodora* extract were fed to intensively reared hares (*Lepus europaeus Pall*.) to evaluate the effects of VB on growth performance and meat quality parameters [78]. Growth performance, carcass weight, and the chemical composition of *Longissimus lumborum* (LL) muscle were unaffected by dietary supplementation with plant extract. The LL cholesterol content was lower (−31.8%) in groups fed the high dosage of VB (10 mg/kg of feed) than in the control. Even if, in groups fed the high dosage of VB, the fatty acid composition revealed an increased content of polyunsaturated fatty acid and omega 3 fatty acids, the resulting oxidative stability was higher than the controls. In fact, the resulting LL muscle vitamin A and E (+37.5% and +69.2% compared to the control) content was higher in groups fed the high dosage of VB, with a positive effect on the meat shelf life.

The data on hares showed that dietary supplementation with plant extract, containing VB, is able to improve blood parameters and systemic antioxidant status, reducing oxidative stress markers and improving health. Moreover, the meat from animals fed a VB-rich extract is healthier due the lower cholesterol content and the high vitamin A and E content, with a positive effect on muscle oxidative stability.

#### 3.1.3. Rabbit

In rabbit, dietary plant extract containing VB was employed to improve health through improvements in the antioxidant status and haematochemical parameters. It was reported that in adult immobilized New Zealand white rabbits, the administration of VB (0.8 mg/kg body weight) twice a day for 21 days, decrease oxidative stress parameters and improved the membrane fluidity of red blood cells [79].

A further study reported that supplementation of rabbits with phytoextract containing VB can be used for the healthy effect on blood parameters and plasma antioxidant status, with a helpful effect on the health and welfare of animals [80]. In fact, a lower plasma concentration of oxidative stress markers and a high vitamin E and A concentration was observed, as reported in Table 2.

Moreover, a reduction in the blood LDL cholesterol (−25.5% than control), bilirubin levels and an improvement of HDL cholesterol (+13.8% than control) and AST liver enzymes was observed.

In a study by Palazzo et al. [81], weaned New Zealand white rabbits were fed with different dosages of *Lippia citriodora* leaves, containing 5 or 10 mg/kg of feed of VB, for 55 days. Growth performance and carcass characteristics were unaffected by dietary treatments. An enhancement of LL muscle lightness during storage was observed, which resulted in a positive effect for the consumers [82]. As reported for hares, the fatty acid profile was influenced by dietary treatments and in both supplemented groups a decrease in saturated fatty acids and an increase in polyunsaturated and omega 3 fatty acids was observed. Moreover, sensory evaluation revealed that the highest dosage of plant extract containing VB enhanced meat tenderness and juiciness.

Dietary supplement with *Lippia citriodora* extract, standardized in 5 mg/kg of feed of VB, was fed to weaned rabbits (White New Zealand × Californian) for 80 days [83]. The results showed an improvement in the oxidative stability of the LL muscle and a positive effect on muscle vitamin A and E content and fatty acid profile, in line with previous results [81].

**Table 2:** Effect of dietary verbascoside on antioxidant parameters in rabbits.

Supplement	Dose mg/kg Feed	Animal	Effect on Antioxidant Parameters	Treatment vs. Control, %	Ref.
*Verbenaceae* extract (*Lippia Citriodora*)	5	New Zealand White × Californian rabbit 80 d	Improved of blood		[80]
			TBARS	−41.04 **	
			ROMs	−31.71 **	
			Vitamin A	+65.98 **	
			Vitamin E	+92.18 **	
*Verbenaceae* extract (*Lippia Citriodora*)	5 10	New Zealand White rabbit 80 d	Improved of muscle		[81]
			TBARS (d 7)	−37 **	
				−34 **	
			Vitamin E	+21.42	
				+71.42	
			(*p* = 0.07)		
*Verbenaceae* extract (*Lippia Citriodora*)	5	New Zealand White × Californian rabbit 80 d	Improved of muscle		[83]
			TBARS (72 h)	−47.73 **	
			Vitamin A	+76.74 *	
			Vitamin E	+30,332 *	

TBARS, thiobarbituric acid reactive substances; ROMs, reactive oxygen metabolite; * *p* < 0.05; ** *p* < 0.001.

#### 3.1.4. Equidae

Natural extract from *Lippia citriodora* containing VB was also used for 6 months as a dietary supplement (0.5 or 1.0 mg of VB/kg metabolic body weight—LW^0.75^) in Avelignese horses to evaluate its effect on blood parameters and antioxidant status [84]. The oxidative stress marker plasma concentration was lower (TBARS −57% compared to the control) in horses fed a VB-rich extract with a high vitamin E and A (+93.9% and +95.8% compared to the control) plasma concentration. A positive impact on blood parameters was also observed with a reduction in blood triglycerides, total and LDL cholesterol, and transaminases.

The same results on blood biochemical parameters and oxidative status markers were observed in lactating jennies of the Martina Franca breed fed a VB-rich extract. The effects of dietary supplementation with plant extracts containing VB on donkey milk fatty acid composition and A and E vitamin levels was also evaluated [85]. The VB-rich extract supplementation improved the milk’s nutritional composition, decreasing saturated fatty acid content and increasing the monounsaturated fatty acid content. An increase in blood vitamin A and E (+39.7% and +26.5% compared to the control) values was also reported.

Dietary plant extract rich in VB was also administered in weaned male Avelignese horses and Martina Franca donkeys [86]. The animals were fed 0.5 mg/kg of metabolic weight (LW^0.75^) of VB-rich extract for 6 months. At 12 months of age, the animals were slaughtered and LL muscle quality parameters were evaluated. The meat color indexes and LL nutritional parameters were unaffected by dietary treatments in both species. In the donkeys, LL muscle oxidative phenomena during refrigerated storage at 4 °C were reduced (−56.2% compared to the control) by the dietary inclusion of plant extracts. The oxidative stability of the LL muscle of Avelignese horses showed no difference in relation to dietary treatments. Probably the dosage of plant extract was unable to reduce oxidative phenomena in horse muscles, which have a high content of polyunsaturated fatty acids compared with donkey muscle [87,88]. The sensory profile of the LL muscle of Martina Franca donkeys showed that red color, typical aroma, sweetness, and tenderness were higher and fibrousness was lower in animals fed a VB-rich extract, compared to controls. This is probably due to the antioxidant activity of the plant extract containing VB, with a stabilizing effect on color parameters and protection against oxidation of *μ*-calpain and m-calpain that positively affect meat tenderness. In Avelignese horses, the sensory descriptors related to typical aroma, saltiness and tenderness were higher and fibrousness was lower in animals fed a VB plant extract, compared to controls. Sensory evaluation revealed that dietary supplementation with a VB-rich extract in both donkey and horse positively affected LL aroma and texture descriptors.

These data suggest the positive effects of dietary VB plant extract in supporting the systemic antioxidant status in *Equidae*, positively modifying blood and hepatic lipid markers. Moreover, the interesting data on meat quality parameters should be expanded in order to better define the VB-rich extract dosage and length of dietary supplementation.

#### 3.1.5. Poultry

Dietary VB from *Verbenaceae* extract was also used as dietary supplement in 42-day-old male broilers (Ross 708). The animals were fed with two dosages of VB (2.5 and 5 mg of VB/kg of feed) for 35 days. Dietary supplementation did not affect growth performance and in vivo antioxidant status. As expected, the nutritional parameters of breast muscle were unaffected by dietary VB, but an improvement of *Pectoralis major* muscle oxidative stability (TBARS −55% compared to the control) was observed [89].

The effects of three levels of *Lippia citriodora* leaf powder (0.25, 0.5, and 1.0%) was also investigated in male broilers (Ross 708) for 42 days [90]. The highest dosage of *Lippia citriodora* dietary supplementation in the broilers positively affected the lipid blood profile (cholesterol −8.7%; Triglyceride –8.82% compared to the control) and increased white blood cells. The *Lippia citriodora* leaf powder, containing VB, positively affected average daily gain, feed intake, and feed conversion ratio in the grower and finisher phases and whole period. The main active components of *Lippia citriodora* are polyphenols, in particular VB, that improve gut health in poultry, with a stabilizing effect on gut microbiota and a positive effect on productive parameters [90]. Broilers fed 1% of *Lippia citriodora* leaf powder had a significantly heavier carcass and breast weight than other experimental groups. Moreover, an improvement of meat quality parameters was also observed due to *Lippia citriodora* leaf supplementation with a higher oxidative stability and lower cooking losses than controls.

The integration of *Lippia citriodora* leaf powder (0.5 and 1.0%) in the feed of broilers exposed to heat stress (a temperature of 35 °C for 8 h daily) from 25 to 42 days of age was studied [91]. At the highest dosage, *Lippia citriodora* leaf powder improved average weight gain and feed intake, reducing the feed conversion ratio, compared to the control group. The breast and bursa weight of Fabricius was higher in animals fed the highest dosage of herbal powder. Moreover, an improvement of serum glutathione peroxidase levels (+51.81% compared to controls) was observed, that could lower the adverse effects of heat stress.

Dietary supplementation with *Lippia Javanica* leaf meal was also included in broilers at 0.25% and 0.5% for 13 weeks. At the high dosage, an improvement of carcass weight was observed. Moreover, an improvement of drip losses and tenderness was also observed [92]. Another study examined dietary inclusion in broiler chicken diets at 0.5% or 1.2% for 42 days [93]. With the lower dosage, a better than average daily gain, feed conversion, and slaughter weight were observed. A positive influence on meat fatty acid composition was also observed with an increase in polyunsaturated fatty acid content and omega 3-fatty acid content.

The same *Lippia Javanica* leaf meal was included in the diet of Japanese quail at 0.25% for 4 weeks and was compared with a diet containing antibiotics [94]. The inclusion of *Lippia javanica* in the quail diet showed no difference in growth performance, health, and meat quality characteristics when compared with the positive control containing antibiotics. A previous study reported that *Lippia Javanica* leaves contained about 1.5 mg of VB/g o dry weight [95].

Moreover, dietary supplementation with 0.1% of *Plantago asiatica* ethanol extract, containing VB, can reduce fecal oocyst shedding and body weight loss in chickens infected with *Eimeria tenella* oocysts, showing an anticoccidial property [96].

These data support the positive effects of *Lippia citriodora* extract and leaf powder in supporting the health and growth performance of broilers, positively affecting antioxidant status and oxidative stability in the meat. Additionally, this dietary supplement seems to be interesting for lowering oxidative stress and supporting productive parameters during heat stress conditions.

#### 3.1.6. Fish

Aquatic animal diseases are one of the main problems for aquaculture, as are the intensification of systems and climate change, promoting the development of infectious diseases [97]. So, in aquaculture, new sustainable strategies are also needed for the treatment of fish diseases, to lower the use of antibiotics and other veterinary drugs [98].

The effects of three levels of *Aloysia citrodora* leaf powder (0.25, 0.5, and 1.0%) were investigated in Rainbow trout (*Oncorrhyncus myskiss*) fingerlings for 6 weeks [99]. The average VB content of the *Aloysia citrodora* leaves was 2.25% [100]. Dietary supplementation with the high dosage of *Aloysia citrodora* leaf powder enhanced serum and skin mucus lysozyme activity and total immunoglobulin levels. Moreover, an improvement in the activity of antioxidant enzymes, such as superoxide dismutase, glutathione-S-transferase, and glutathione peroxidase, was also observed.

The inclusion of 0.1% of leaf extract from *Salvia officinalis* and *Lippia citriodora*, contained 2% of VB, was also evaluated in gilthead seabream (*Sparus aurata*) for 92 days [101,102]. An improvement in growth performance and feed conversion ratio was observed [101]. Moreover, dietary supplementation with leaf extract sustains gut homeostasis and enhances intestinal epithelium integrity [102]. The same VB-rich extract was integrated at 0.1% into the diet of Atlantic salmon (*Salmo salar*) for 133 days. Better growth performance and enhancement of the systemic immune response were observed in the group fed the VB-rich extract. In the same experimental group, a lower mortality was recorded in fish submitted to bacterial challenge with *Aeromonas salmonicida*, which causes furunculosis in salmonids [103]. These data suggests that VB-rich extracts may be considered as a promising sustainable additive for aquafeeds.

### 3.2. Ruminants

#### Small Ruminants

The effect of *Plantago lanceolata* (36 mg of VB per g of dry matter) was tested in an in vitro assay, with cow rumen fluid, to assess its potential to influence ruminal fermentation. The results suggested that VB enhanced rumen fermentation and reduced ammonia production. These effects may be due to use of VB as an energy source, with an increase in fermentable carbohydrates that may reduce nitrogen loss from ruminants [104].

Dietary supplementation with a water-soluble extract of *Verbenaceae* (*Lippia* spp.) leaves containing VB was also studied in ewes and lambs. The effects of dietary extruded flaxseed (200 g/kg feed) and water-soluble extract of *Verbenaceae* (*Lippia* spp.) leaves containing VB (2.86 g/kg feed) or VB rich extract plus vitamin E (14.29 g/kg feed) over 40 days of *post-partum Lacaune* ewes was evaluated [105]. An improvement of blood antioxidant status (Vitamin E +233.9% compared to the control; Vitamin A +50.3% compared to the control; TBARS −71.3% compared to the control; ROMs −58.8% compared to the control) and lipid blood parameters (triglycerides −29.6% compared to the control; total cholesterol −4.6 compared to the control; HDL cholesterol +28.9% compared to the control) were observed after VB supplementation without additional health effects related to vitamin E dietary integration. In a study of suckling lambs supplemented with two different dosages of *Syringa vulgaris* extract (2.5 or 5 mg/d of VB) for 42 days, significant improvements in body weight, average daily gain, and milk consumption were observed [106]. An improvement of plasma oxidative stress markers and lipid blood parameters was also reported. In ovine species, dietary supplementation with VB improved productive parameters and decreased oxidative stress markers in the immediate postpartum period and in suckling lambs.

Dietary *Lippia alba* hay was integrated into the diet of Alpine goats at 0, 30, 60, and 90 g/kg of total dry matter for 13 days [107]. A previous study reported that VB was the major metabolite detected in *Lippia alba*, even if seasonal variation was observed [108]. The dry matter intake increased with *Lippia alba* hay inclusion. It is possible that this effect may be linked to the ability of *Lippia alba* to modulate the rumen microbial activity [109]. Moreover, milk yield and fat were improved in goats fed with *Lippia alba* without effects on hematological and rumen parameters.

**Figure 2:**
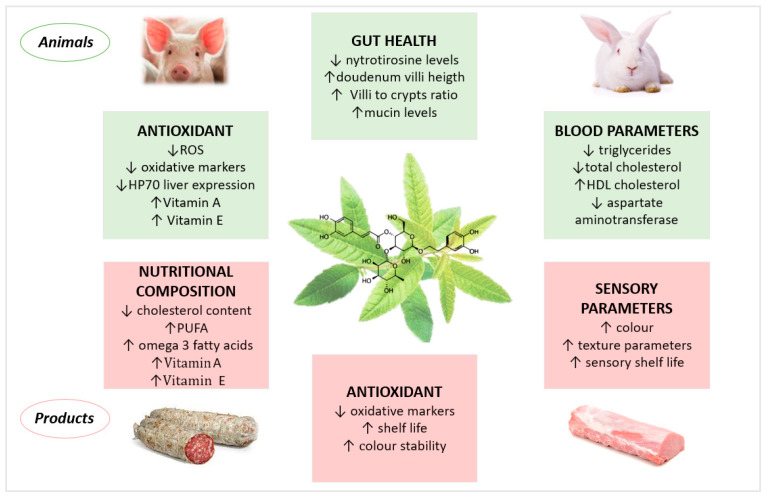
Biological activities of *Lippia citriodora* extracts containing verbascoside in livestock: effects on in vivo parameters and on meat and product quality.

The effects of dietary supplementation with *Lippia citriodora* extracts, containing verbascoside in livestock, on in vivo parameters and on meat and product quality is reported in Figure 2.

## 4. Conclusions

The numerous biological activities of VB could be used to improve the productive performance, health parameters, and antioxidant status in livestock. The antioxidant activity of natural extracts containing VB may have valuable effects in supporting systemic and gut antioxidant status, reducing the negative effects of oxidative stress, and modulating gut health. A positive effect of supplementation with VB-rich extracts on lipid blood parameters has also been reported. Moreover, dietary supplementation with VB plant extracts positively affects meat and product quality parameters, enhancing muscle vitamin content with a positive effect on oxidative and color stability and product shelf life. The positive effects of dietary supplementation with VB-rich extracts on fatty acid composition and texture indexes, such as tenderness, have been also observed. In conclusion, VB-rich extracts seem to be a promising natural supplement in livestock diet, helping to sustain health, antioxidant status, and product quality. Further research is needed to deepen understanding of the mechanism of action of plant extracts containing VB in several organs and tissues, optimizing the dosages in both monogastric and ruminant species.

## Figures and Tables

**Table 1:** Phenylpropanoid glycoside and benzoic acid content of *Lippia citriodora* extract.

Compounds	g/kg Extract
Gallic acid	1.755 ± 0.07
3.4-dihydroxybenzoic acid	0.450 ± 0.04
Methyl gallate	1.955 ± 0.09
Isoverbascoside	0.455 ± 0.04
Verbascoside	4.470 ± 0.08

## Data Availability

Data will be provided upon request.
